# Malignant transformation of WHO grade I meningiomas after surgery or radiosurgery: systematic review and meta-analysis of observational studies

**DOI:** 10.1093/noajnl/vdaa129

**Published:** 2020-10-16

**Authors:** Satoshi Nakasu, Akifumi Notsu, Kiyong Na, Yoko Nakasu

**Affiliations:** 1 Division of Neurosurgery, Kusatsu General Hospital, Kusatsu, Japan; 2 Department of Neurosurgery, Shiga University of Medical Science, Ohtsu, Japan; 3 Clinical Research Center, Shizuoka Cancer Center, Nagaizumi, Japan; 4 Department of Pathology, Kyung Hee University Hospital, Kyung Hee University College of Medicine, Seoul, South Korea; 5 Division of Neurosurgery, Shizuoka Cancer Center, Nagaizumi, Japan

**Keywords:** incidence, malignant transformation, meningioma, meta-analysis, radiosurgery

## Abstract

**Background:**

The incidence and clinical features of the malignant transformation of benign meningiomas are poorly understood. This study examined the risk of the malignant transformation of benign meningiomas after surgery or stereotactic radiosurgery.

**Methods:**

We systematically reviewed studies published between 1979 and 2019 using PubMed, Scopus, and other sources. We analyzed pooled data according to the PRISMA guideline to clarify the incidence rate of malignant transformation (IMT) and factors affecting malignant transformation in surgically or radiosurgically treated benign meningiomas.

**Results:**

IMT was 2.98/1000 patient-years (95% confidence interval [CI] = 1.9–4.3) in 13 studies in a single-arm meta-analysis. Although the evidence level of the included studies was low, the heterogeneity of the incidence was mostly explained by the tumor location. In meta-regression analysis, skull base tumors had a significantly lower IMT than non-skull base tumors, but no gender association was observed. IMT after radiosurgery in 9 studies was 0.50/1000 person-years (95% CI = 0.02–1.38). However, a higher proportion of skull base tumors, lower proportion of males, and lower salvage surgery rate were observed in the radiosurgery group than in the surgery group. The median time to malignant change was 5 years (interquartile range = 2.5–8.2), and the median survival after malignant transformation was 4.7 years (95% CI = 3.7–8) in individual case data.

**Conclusion:**

IMT of benign meningioma was significantly affected by the tumor location. Radiosurgery did not appear to increase IMT, but exact comparisons were difficult because of differences in study populations.

**Key Points**

- IMT after surgery for benign meningioma was 2.98/1000 person-years.
- A skull base tumor location strongly affected IMT.
- IMT after radiosurgery was influenced by its specific patient background.

**Importance of the Study**

Some benign meningiomas recur as higher-grade lesions. Although their prognosis is reportedly worse than that of de novo high-grade meningiomas, the incidence and clinical features of such aggressive grade I meningiomas are unknown. We conducted a meta-analysis and systematic review and found that the incidence rate was 2.98/1000 person-years. The rate was influenced by the tumor location. The low rate after radiosurgery (0.5/1000 person-years) is probably attributable to the high proportion of skull base tumors and low rate of salvage surgery after progression.

Meningiomas are usually indolent tumors that often remain asymptomatic.^1^ Even when symptomatic, approximately 80% of meningiomas are categorized as WHO grade I and slow-growing tumors that exhibit decelerated growth during enlargement.^2^ However, even benign meningiomas can recur after total removal, and some of these lesions are known to behave more malignantly. The prognostic factors of benign meningiomas have been investigated by several authors. The extent of removal is the most important factor,^3–5^ and tumor proliferative potential, tumor location, and gender are probable candidates.^6–9^

Atypical or anaplastic meningiomas are likely to recur, and they are associated with poor clinical courses. High-grade meningiomas have significantly elevated rates of chromosomal gains and losses, especially among tumors with monosomy 22,^10^ and they carry a higher mutation burden than low-grade tumors. DNA methylation patterns may serve as diagnostic biomarkers for malignancy.^11^ It is reported that approximately 20–40% of meningiomas are secondary tumors that originated from WHO grade I tumors.^12–14^ Reports in the literature indicate stepwise genetic progression, with the deletion of chromosome 22 representing the fundamental alteration and deletions in other chromosomes (1p, 14q, and 10q) occurring during the progression of these tumors toward a more malignant type.^15–17^ Consequently, higher-grade meningiomas are categorized as de novo or secondary tumors.^18^ There may be genetic differences between these 2 types; specifically, promoter mutation of hTERT is sometimes found in the latter but rarely detected in the former.^18–20^ However, the differences between de novo and secondary higher-grade meningiomas are unclear.

It has been reported that 1–2% of benign meningiomas transform into higher-grade lesions, but these data were based on the old pathological grading system prior to the release of WHO 2000.^21,22^ Schiffer et al.^23^ claimed that malignant transformation was extremely rare, observing transformation to an atypical lesion in only 2 of 53 cases of recurrence of initially benign meningioma according to the WHO 2000 criteria. Conversely, McGovern et al.^24^ reported that 8 tumors progressed to atypical or anaplastic lesions during recurrence among 175 benign tumors based on the WHO 2000 criteria. Yeon et al.^25^ and Champeaux et al.^4^ reported that 2.7% and 2.2% of benign meningiomas exhibited malignant progression based on the WHO 2016 criteria, respectively. It is unclear whether these differences are based on the differences of the pathological criteria or other factors such as changes in treatment modalities and the increased use of radiosurgery. For rare outcomes, meta-analysis may be the only method for obtaining reliable evidence.

Stereotactic radiosurgery (SR) is a treatment option for patients with recurrent or surgically challenging residual tumors. A major concern of SR is the expected risk of new malignancy or malignant transformation. Fractionated radiotherapy is well known to be related to oncogenesis.^26^ Meningiomas previously treated with adjuvant radiation exhibit a significantly higher frequency of copy number alterations than radiation-induced or radiation-naïve meningiomas.^10^ However, a recent multicenter cohort study reported no malignant transformation among 1490 benign meningiomas treated with SR during a median follow-up period of 8.1 years.^27^ Conversely, other studies reported a relatively higher transformation rate in meningiomas treated with SR.^28,29^ The effect of SR on malignant transformation in meningiomas is thus uncertain.

Because of its rarity, the incidence and clinical features of the malignant transformation of benign meningiomas are poorly understood. We systematically reviewed previously reported cases of initially benign meningiomas that displayed malignant transformation. We estimated the incidence rate of malignant transformation (IMT) because the percentage of malignant transformation (PMT) may increase with longer follow-up. We examined whether known prognostic factors of benign meningiomas (extent of removal, location, and gender) were associated with malignant transformation. Individual case data were analyzed to identify the clinical features of this aggressive tumor. Another aim of this study was to compare the frequency of malignant transformation between surgical and radiosurgical series.

## Materials and Methods

### Literature Search and Data Extraction

This study followed the PRISMA statement (Supplementary File 1). The search flow diagram is outlined in Supplementary Figure 1. We used the keywords “meningioma” and “recurrence” combined with “human” to search for studies published in English or Japanese from 1979 to December 2019 in PubMed, Scopus, Cochrane Library, and Google Scholar. In addition, we searched in Japan Medical Abstract Society. “Malignant transformation,” “malignant change,” “malignant progression,” and dedifferentiation were the combined keywords. Studies confined to neurofibromatosis, pediatric studies, and studies of restricted tumor locations were excluded. Studies of spinal, optic nerve, and intraorbital meningiomas were also excluded. Two of the authors searched the literature independently, and the final selection was performed via discussion.

Because the majority of the selected studies reported outcomes for patients without focusing on “malignant transformation,” they often lacked necessary data. We attempted to obtain the data by contacting the authors of each study. Four of the authors kindly responded to our e-mails, and 3 authors provided the necessary data.^5,25,30^

### Risk of Bias

As all analyses were retrospective observational studies that described the rate of malignant transformation for benign meningiomas, we did not use a specific method to assess the risk of bias. Instead, we carefully investigated the heterogeneity of the studies. Publication bias was assessed in analyses including more than 10 studies.

One possible bias was that each surgical study employed different WHO pathological criteria. Meanwhile, the initial diagnosis was not available for a median of 40.8% (interquartile range [IQR] = 33.0–46.8) of patients in the SR group. We evaluated these factors in statistical analysis.

### Extraction of Incidence Data

We included studies that described at least 50 cases of WHO grade I meningioma with suitable follow-up periods and the rate of malignant transformation at the time of recurrence. IMT was calculated as the number of transformations per 1000 person-years. Separately, PMT was calculated in studies with a mean follow-up period exceeding 5 years. In each study, PMT was defined as the number of observations of malignant transformation divided by the number of all observations. As PMT did not account for the observational time, this variable was difficult to interpret statistically. However, if the rate of malignant transformation of meningioma plateaus during a long observation time, PMT is a more appropriate outcome than IMT.

When only the median follow-up data were available, means were calculated using the following equation obtained from 11 studies that described both mean and median values (*R*^2^ = 0.95): mean = 1.13 × median.

In each study, we collected the median or mean age at initial presentation and follow-up period, sex distribution, and the numbers of WHO grade I benign meningiomas, recurrences, and malignant transformations. The tumor location was categorized as a skull base (SB) and a non-skull base (NSB). When the exact data for tumor location or gender were available for all-grade meningiomas but not for grade I exclusively, the former data were used to estimate the rate of SB tumors and the proportion of males. We also assessed the rate of surgical treatment before SR and at the time of recurrence or progression.

### Extraction of Individual Case Data

We selected the data of individual cases of benign meningiomas with a malignant transformation from articles identified during the literature search. The selected articles included both research and case reports. In this analysis, we extracted individual data for initial age, sex, tumor location, time to malignant change, number of recurrences before the malignant change, receipt of radiation therapy before and after malignant transformation, survival after malignant transformation (SMT), initial histological findings, and initial radiological findings on CT or MRI when available. We collected data regarding the existence of perifocal edema, tumor shape (lobulated or round), and homogeneity after contrast enhancement as radiological findings and the subtype of meningiomas and existence of mitoses as histological findings.

### Pathological Diagnosis

The WHO grading system for brain tumors was first published in 1979,^31^ and the criteria have been revised several times. Because the initial WHO grading system of meningiomas included hemangiopericytic and hemangioblastic types, we excluded them from the analyses. The distinction of atypical meningiomas was not described until the WHO 1993 grading system,^32^ whereas many studies distinguished this aggressive subtype after the publication of studies by Jellinger et al.^21^ and Jääskeläinen et al.^22^ Meningioma grading criteria were substantially revised in 2000.^33^ At this time, brain-invasive but otherwise benign meningiomas were not categorized as atypical meningiomas, but they were described as aggressive tumors with the same biological activity as atypical meningiomas. The 2007 classification defined that brain-invasive but otherwise benign meningiomas should prognostically be considered WHO grade II lesions,^34^ but the WHO 2016 criteria classified these tumors as atypical lesions.^35^ In statistical analyses, we categorized the WHO criteria into 3 groups: before 2000, 2000 and 2007, and 2016. When a study using the WHO 2007 criteria classified brain-invasive meningiomas as atypical tumors, it was included to the WHO 2016 group.

In individual case analyses, several case reports or series published before the WHO 2016 classification categorized brain-invasive meningiomas as non-benign tumors. If it was not the case, then we categorized them as atypical meningiomas whenever possible. Some old studies reporting individual case data described precise histological findings about cellularity, loss of architecture, mitosis, necrosis, and brain invasion. We reclassified such tumors according to the WHO 2016 classification.^35^

### Statistical Analysis

We used R statistical software for statistical analyses. Because the selected studies that covered a long period were expected to report heterogeneous data, the random-effects model was applied in the meta-analysis (DerSimonian–Laird method). We used “metarate” in R to perform a single-arm meta-analysis to calculate IMT or PMT. Because several studies reported a null incidence, we applied the Freeman–Tukey double arcsine method for transformation. Publication bias was estimated using a funnel plot when 10 or more studies were included. Linear regression analysis was used in the test for funnel plot asymmetry. The reviewed studies were tested for heterogeneity (Q test, *I*^2^ statistic), and we performed meta-regression analyses to identify factors related to heterogeneity.

For individual data analyses, univariate analysis was conducted via the Fisher’s exact test for categorical variables and the Mann–Whitney U test or Kruskal–Wallis test for continuous variables using EZR in R.^36^ Possible candidate predictors related to survival were included in multivariate regression analysis performed using the Wald method. The Kaplan–Meier method was used in survival analyses. We performed analyses using the log-rank test and Cox proportional hazard test. The correlations of 2 factors were examined using Pearson’s correlation coefficient (*r*).

Two-sided *P* < .05 was considered statistically significant.

### Ethical Approval and Informed Consent

This review did not involve direct studies on humans, and thus, informed consent not was required.

## Results

In the postsurgical series, we selected 13 studies with follow-up data for incidence analysis^4,5,24,25,30,37–44^ and another 2 studies with follow-up periods exceeding 5 years but unknown mean values for percentage analysis^22,45^ (Supplementary Table 1).

In the SR series of meningiomas, we identified 13 studies.^27–29,46–55^ Two large multicenter studies covered the results of some of these studies.^27,29^ Because the 2 multicenter studies contained data from overlapping institutes, we chose a larger study with necessary data for data extraction. Finally, we selected 9 studies for incidence analyses to avoid overlapping data^28,29,47,50–57^ (Supplementary Table 2).

We selected 172 cases from 93 articles for individual case data analysis (Supplementary Table 3). Sixty-five cases were obtained from case reports, and 107 cases were obtained from research articles.

### Malignant Transformation in Surgical Series

**Figure 1.**
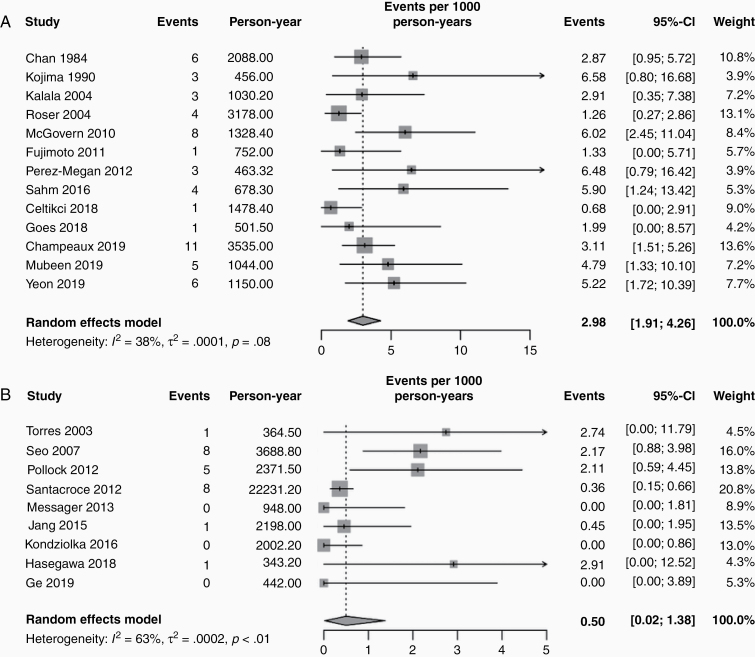
Forest plot showing the incidence rates of malignant transformation in the surgical (A) and radiosurgical series (B).

Thirteen postsurgical series reported 56 cases of malignant transformation among 2639 patients with WHO grade I meningiomas during a median follow-up period of 6 years (IQR = 5.7–8.1, Table 1).^4,5,24,25,30,37–44^ IMT was 2.98/1000 patient-years (95% confidence interval [CI] = 1.9–4.3, test of heterogeneity: *Q* [*df* = 12] = 19.46, *P* = .078, Figure 1A). The funnel plot exhibited slight asymmetry without significance (*P* = .063) in a linear regression test.

**Table 1.** Study Population in Surgery and Radiosurgery Group

	Surgery Group		Radiosurgery Group	
	2639 Patients in 13 Studies		5969 Patients in 9 Studies	
	Total	Median	Total	Median
Gender, Female/male	1050/410 (*n* = 9)	Male rate 27.5%, IQR [26.3–29.7]	1580/502 (*n* = 7)	Male rate 25.4%, IQR [20.5–30.5]
Age (years)	*n* = 12	54.7, IQR [51.7–58.1]	*n* = 8	55.7, IQR [54.1–57.1]
Skull base/non-skull base	625/759 (*n* = 9)	SB rate 41.5%, IQR [38.3–49.4]	1027/325 (*n* = 5)	SB rate 73.7%, IQR [69.8–77.5]
Recurrence or progression	468/2639 (*n* = 13)	18.8%, IQR [9.2–26.4]	464/5969 (*n* = 7)	7.5%, IQR [5.4–9.2]
Follow-up period (years)	*n* = 13	6.0, IQR [5.7–8.1]	*n* = 9	5.7, IQR [3.5–7.1]
Salvage surgery for recurrence	196/388 (*n* = 9)	Rate* 68.4%, IQR [33.3–89.4]	94/455 (*n* = 8)	Rate* 35.9%, IQR [26.2–43.3]
Malignant transformation	56 in 2639 (*n* = 13)	PMT 2.16% (*n* = 14)	24 in 5969 (*n* = 9)	PMT 0.51% (*n* = 6)
Transformation per person-year	56 in 17 683 (*n* = 13)	IMT 2.98/1000 person-year	24 in 34 589 (*n* = 9)	IMT 0.50/1000 person-year

Total, the total number in the studies; median, the median value in the studies; male rate, male number/total number; SB rate, number of skull base tumor/total number; Rate*, number of salvage surgery/number of recurrence or progression; PMT, percentage of malignant transformation; IMT, incidence rate of malignant transformation.

PMT was determined from studies with a mean follow-up period exceeding 5 years. We excluded 1 study with a short follow-up period and included 2 other studies that had apparently follow-up periods exceeding 5 years without specifying the exact duration.^22,45^ According to data from the 14 studies, PMT was 2.16% (95% CI = 1.46–2.96).

**Figure 2.**
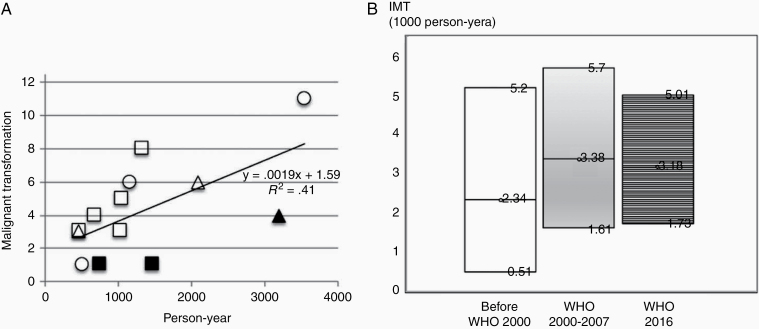
WHO pathological criteria and incidence rate of malignant transformation (IMT). (A) Relationship between the number of malignant transformations and person-years (*R*^2^ = 0.41, *P* = .02). Circle, WHO 2016; square, WHO 2000 or 2007; triangle, before WHO 2000. Filled squares or triangles denote studies in which more than 50% of lesions were skull base tumors. (B) IMT did not differ among the WHO grading systems (*P* = .695). The incidence rate and 95% confidence interval are shown.

When transformation numbers were plotted against person-years in each study, a significant correlation was found (*R*^2^ = 0.41, *P* = .02, Figure 2A) despite the use of different WHO grading systems. Studies with a high SB tumor rate (>50%) reported a low transformation rate (Figure 2A). If 3 studies with an SB tumor rate exceeding 50% were excluded, the correlation became much stronger (*R*^2^ = 0.77, *P* = .0009).

### Factors Related to Malignant Transformation

We analyzed contributing factors to IMT via meta-regression. IMT did not differ by WHO grading system (test for residual heterogeneity: *Q* [*df* = 2] = 0.728, *P* = .695, Figure 2B).

**Figure 3.**
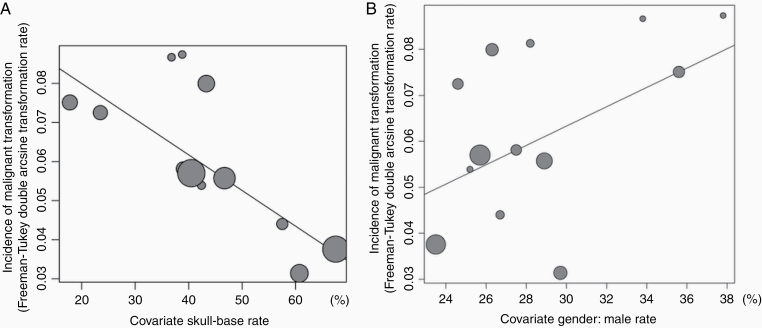
Meta-regression of the incidence rate of malignant transformation (IMT) in surgical series by tumor location (A) and gender (B). A skull base location significantly affected IMT (*P* = .0005), whereas gender had no effect (*P* = .088). Size of circle = weight.

Meta-regression analysis revealed that an SB tumor location significantly affected IMT (*Q* [*df* = 10] = 5.889, *P* = .0005, Figure 3A), whereas the relationship between gender and IMT did not reach significance (*P* = .088, Figure 3B).

The relationship between the extent of initial surgical resection and the frequency of malignant transformation was available in only 3 studies.^24,39,42^ We were unable to evaluate the relationship because these studies lacked time-scale data, whereas meta-analysis of odds ratios (ORs) did not reveal a significant effect (*P* = .21).

### Malignant Transformation After SR

Nine studies including one large multicenter retrospective studies reported 24 cases of malignant transformation among 5969 patients (Table 1).^28,29,47,50–55^ Of these, 3062 (51.2%) patients underwent surgery before SR, whereas a pathological diagnosis was not available for the other patients. When transformation numbers in each study were plotted against person-years, the correlation was poor (*R*^2^ = 0.30, *P* = .16). The incidence was approximately 0.50/1000 person-years (95% CI = 0.02–1.38, Figure 1B).

Remarkably, SR studies displayed high heterogeneity concerning IMT (*I*^2^ = 63%). We performed a meta-regression analysis of location, gender, and the rate of salvage surgery after progression (salvage surgery/total treated patients). However, we could not identify the cause. Only the rate of salvage surgery displayed borderline significance (*P* = .079). We were unable to compare the difference by the WHO grading system because of the lack of data.

PMT was determined from 6 studies with mean follow-up periods exceeding 5 years as 0.51% (95% CI = 0.18–1.41, Table 1). Although both IMT and PMT were much lower in the SR group than in the surgery group, the features of the populations were completely different between these groups (Table 1). Patients in the SR group had a higher rate of SB tumors, a higher proportion of female patients, and a lower likelihood of surgery after tumor progression.

According to imaging, 40–70% of lesions in the SR series were presumably benign meningiomas. Because the majority of studies presented no cases of “malignant transformation without initial surgery,” such cases might have been excluded from the studies. Therefore, we separately analyzed patients who underwent surgery before radiosurgery. IMT in this group was 0.82/1000 patient-years (95% CI = 0.0–3.02). The rate of malignant transformation was determined from 6 studies with a mean follow-up period exceeding 5 years as 1.11% (95% CI = 0.00–3.58).

### Individual Case Analyses

One hundred seventy-two cases were analyzed (Supplementary Table 3), including 92 females and 80 males (Table 2). The median age at initial diagnosis was 52 years. The majority of meningiomas transformed at the first (62%) or second recurrence (27.2%), whereas transformation after the third recurrence was less common. SB tumors (*n* = 41) were far less common than NSB tumors (*n* = 104). Transformed SB tumors were more frequently observed in female patients than in male patients (29 vs 12, *P* = .038), whereas the number of NSB tumors was similar between females and males (51 vs 53). Pathological and radiological findings were rarely obtained. Meningotheliomatous meningioma was the most frequent subtype at the initial pathological diagnosis. Most fibrous (14/15) and transitional meningiomas (19/23) were NSB tumors, whereas 35.6% of meningotheliomatous meningiomas (21/59) had an SB location (*P* = .04).

**Table 2.** Individual Case Data: Initial Clinicopathological Features

	*N* = 172		
Gender	Female 92, Male 80		
Initial age (years)	Median 52, IQL [41–60] (*n* = 171)		
	Mean 50.9, SD 13.4		
Time to malignant change (years)	Median 5 (*n* = 154), IQL [2.5–8.2]		
	Mean 6.6, SD 6.0		
Location	*Non-skull base 104*		
	Convexity 43	Parasagittal 37	
	Falx 12	Ventricle 5	NA 7
	*Skull base 41*		
	Frontal base 2	Olfactory groove 3	
	Cavernous 3	Sphenoid ridge 16	
	Middle fossa 2	Tentorial 7	
	CPA 4	Petroclival 2	NA 2
	*NA 27*		
Degree of initial resection	Total (Simpson 1–3) 86		
	Subtotal (Simpson 4) 32		
	NA 54		
Initial histology	Meningo 61	Fibrous 15	Transit 25
	Psamm 1	Secretory 1	NA 69
	Mitosis (+) 17	Mitosis (−) 19	NA 136
Initial radiological features	Edema (+)16 (−)5		
	Lobulated shape (+)20 (−)5		
	Heterogenous CE (+)14 (−)16		
Published year	After/before 2001, 113/59 (before 1993, 25 cases)		

CPA, cerebellopontine angle; CE, contrast enhancement; NA, not available.

No differences were found concerning the initial age at diagnosis, sex, location, and extent of resection between studies published before and after 2001. However, patients described in studies published in 2001 or later more frequently underwent radiation therapy (including SR) before malignant transformation (38/99 vs 2/54, *P* = .0001) than those described in earlier studies, and they more commonly had WHO grade II (61 vs 21, *P* = .025).

### Time to Malignant Transformation

**Figure 4.**
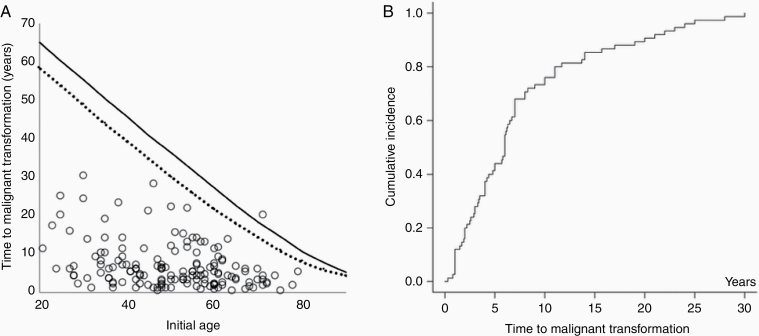
(A) Relationship between the initial patient age and time to malignant change in individual case data. Life expectancy curve in 2000 in Japan: solid line, women; dotted line, men. (B) Cumulative incidence of malignant transformation in patients ≤50 years old from individual case data. The incidence increased linearly in the initial 8–9 years.

Median time to malignant transformation (TimeMT) was 5 years (IQR = 2.5–8.2). Tumors in younger patients had a longer TimeMT (*r* = −0.33, *P* = .032). In univariate analyses, TimeMT was significantly longer in patients who received radiotherapy before malignant transformation than in patients who did not receive radiotherapy (6.6 years vs 4.0 years, *P* = .006), whereas no differences were observed according to the extent of tumor removal (*P* = .42), sex (*P* = .09), or tumor location (*P* = .56). Multivariate analyses identified younger age as the only factor associated with longer TimeMT (*P* = .004). However, a scatter plot indicated that TimeMT was apparently limited by life expectancy in elderly patients (>50 years old, Figure 4A).

A primary study aim was to determine IMT. However, no reports described the time course of the transformation rate. For this purpose, we created a cumulative incidence curve of malignant transformation in younger patients (≤50 years old, *n* = 56). The curve revealed an almost linear increase in the first 8–9 years and a slower increase thereafter (Figure 4B).

### Survival After Malignant Change

SMT was calculated within 30 days after surgery. Overall, median SMT was 4.7 years (95% CI = 3–7.8). Factors related to longer SMT were younger age (≤55 years, *P* = .032) and last WHO grade diagnosis (*P* = .003; Table 3). Tumor location (*P* = .63), gender (*P* = .64), and radiation therapy after progression (*P* = .09) were not prognostic factors. SMT did not differ by WHO grading system (*P* = .38) or type of publication (case report or research article, *P* = .09). Multivariate analyses illustrated that the WHO grade (grade II [median, not reached; 10-year overall survival (OS), 50.1%, 95% CI = 23.4–72.0] vs grade III [median, 3.1 years] or grade III after grade II [median, 4.1 years]) was the only significant prognostic factor (*P* = .012)

**Table 3.** Survival After Malignant Transformation

Factors	Survival Median Years	Log Rank	Multivariate Cox Proportional Hazard Test
Gender	Female 4.0 (*n* = 63)	*P* = .64	
	Male 4.3 (*n* = 46)		
Age at malignant transformation	55 or less, 6.3 (*n* = 49)	*P* = .032	*P* = .10
	Older than 55, 3.5 (*n* = 57)		
Last WHO grade	Grade II (*n* = 45)	*P* = .007	II vs III (including III after II)
	NR [4.2–NR]		
	Grade III (*n* = 43)		*P* = .012
	3.1 [2.4–4.7]		
	Grade III after II (*n* = 21)		HR 2.45, 95% CI [1.22–4.93]
	4.1 [1.7–6.3]		
Location	Skull base 3.0 (*n* = 25)	*P* = .63	
	Non-skull base 4.2 (*n* = 69)		
Radiation after recurrence	Yes 4.3 (*n* = 60)	*P* = .09	*P* = .09
	No 3.0 (*n* = 25)		
Published year	2000 or before 3.7 (*n* = 46)	*P* = .38	
	After 2001 4.6 (*n* = 63)		

CI, confidence interval; NR, not reached; radiation, radiation therapy including fractionated local radiotherapy and radiosurgery; HR, hazard ratio.

## Discussion

In this systematic review and meta-analysis, we calculated IMT for benign meningiomas in surgically treated patients as 2.98/1000 patient-years. The rate was significantly affected by the tumor location, as SB tumors had a lower transformation rate than NSB tumors. The reported incidence in the SR group was much lower than that in the surgery group. However, the surgery and SR groups consisted of extremely different patient populations regarding tumor location and gender, in addition to differences in patient numbers. Therefore, we must cautiously evaluate the role of radiosurgery in malignant transformation.

### The Rate of Malignant Transformation in the Surgery Group

The rate of malignant transformation for benign meningiomas has been reported to be as low as 1–2%.^21,22^ Recent studies found a wide range of transformation rates (0.30–4.9%) for WHO grade I tumors (Supplementary Table 1). As shown in Figure 4B, malignant transformation occurs more than 20 years after the initial surgery. Thus, studies with longer follow-up periods are expected to have a higher incidence. For this reason, we evaluated the incidence by person-year and found a relatively good correlation between the number of transformations and the duration of follow-up (person-years, Figure 2A). However, the cumulative transformation rate in individual data analysis revealed that malignant transformation may not have constantly occurred. Figure 4B illustrates that the increase was linear in the first 8–9 years before slowing thereafter. The rate of increase may possibly plateau after 30 years. The reason for the slower rate is unclear, although it might be partly attributable to the loss of patients to follow-up. Because of this uncertainty, we do not recommend applying the incidence rate in this study to predict the risk of patients with more than 10 years of follow-up. In this context, PMT (2.16%, mean follow-up >5 years) may possibly increase. Considering median TimeMT in individual data analysis to be 5 years (mean, 6.6 years), PMT might increase up to 4–5% when sufficient longer follow-up data are available.

There was no significant difference in IMT according to the pathological criteria (Figure 2B). Although some “benign” tumors in prior WHO grading systems may have been considered atypical tumors in new grading systems, this appears to be uncommon. Yeon et al.^25^ revealed that 5 of 291 meningiomas that were surgically treated between 1998 and 2013 were reclassified into other grades in the WHO 2016 criteria. Our study reported a higher incidence of transformation to grade II meningioma in studies reported in 2001 or later than in those reported before 2001 in individual data analysis. Some secondary malignant meningiomas (WHO grade III) reported before 2001 might have been grade II tumors. Contrarily, in the surgical series, we did not detect a difference in IMT among the grading systems. Thus, the judgment of histological progression to an aggressive form might not be largely affected by changes in the pathological criteria.

### Tumor Location

Accumulated evidence has revealed differences in meningiomas between locations. NSB meningiomas more frequently had a high grade than SB tumors.^9,56^ The differences may be based on genomic subgroups including *NF2* mutation in NSB tumors, Hedgehog signaling pathway mutation in midline tumors, and non-*NF2* mutation in anterior SB tumors.^57^ Furthermore, Yoshida et al.^58^ reported that the origin of the meninges differed by location; specifically, the meninges of the midbrain and hindbrain had a mesoderm origin, whereas the meninges of the forebrain had a neural crest origin.

McGovern et al.^24^ first reported a higher malignant transformation rate for NSB meningiomas than for SB meningiomas using Fisher’s exact test (*P* = .024). Although no other studies compared the malignant transformation rate by tumor location, we found that the tumor location significantly influenced IMT. In surgical studies, the heterogeneity of IMT was attributable to the proportion of SB tumors. The increase in the number of specialized institutions for SB surgery biased the distribution of the location of meningiomas and influenced IMT in each report. Conversely, the difference of IMT by tumor location in the SR group was not significant because of the uniformly high frequency of SB tumors. Although meta-analyses for malignant transformation in the SR group indicated that an SB location significantly affected the rate of malignant transformation (OR = 0.26, 95% CI = 0.08–0.86, *P* = .027), we were concerned that this analysis did not consider the observation period.

Location is a clinically important factor in the treatment of meningiomas. SB tumors are generally difficult to treat surgically, and consequently, the recurrence rate is high.^59^ Interestingly, recent studies revealed biological differences between SB and NSB tumors. Hashimoto et al.^7^ reported lower proliferation potential for SB meningiomas, although this finding remains controversial. The discrepancy may be partly attributable to differences in the classification of tumor location. Tentorial meningiomas were previously categorized as SB tumors,^24^ whereas Hashimoto et al.^7^ and others classified them as NSB tumors.^59,60^ Although we categorized these lesions as SB tumors, the rate of malignant transformation for tentorial meningiomas was relatively high in individual case data (Table 2). In fact, recent genetic studies demonstrated that tentorial meningiomas had a relatively high *NF2* mutation rate, similarly as observed for NSB tumors.^57^

Male sex had borderline significance for high malignant transformation potential in meta-regression analysis. Of note, Meling et al.^9^ reported that meningiomas more frequently have an SB location in females than in males. Several other authors reported similar trends without statistical significance.^5,61,62^ Therefore, gender differences in the rate of transformation might be influenced by the tumor location.

### TimeMT and Survival in Individual Data

Median TimeMT in individual case data was 5 years (mean, 6.6 years). The value was comparable with that reported in studies that were not included in this analysis. Champeaux et al.^12^ reported in their study of 194 atypical meningiomas that 31 benign meningiomas progressed to grade II meningioma after a median of 5.7 years (IQR = 2.1–13). Yang et al.^14^ analyzed atypical and anaplastic meningiomas, observing that 17 of 64 high-grade meningiomas had transformed from benign lesions. They found mean TimeMT from benign to atypical was 70.0 months, and that from benign to anaplastic was 39.8 months. Krayenbühl et al.^63^ reported a mean value of 13.8 years (range. 0.25–24) among 10 secondary atypical meningiomas. However, no study examined the changes in the rates. Our analysis of individual case data revealed a linear increase in the first 8–9 years with a slower rate thereafter (Figure 4B). Because information is limited because the data were compiled from case reports or series, further study is necessary for reaching a definitive conclusion.

SMT in patients with secondary high-grade meningioma is known to be shorter than OS among patients with de novo high-grade meningiomas.^12,63–65^ Mean SMT in patients with secondary anaplastic meningioma has been reported as 1.1–2.1 years.^63,64,66^ The values were slightly smaller than those in our study (median, 3.1 years) because the former studies included initially grade II meningiomas. In patients with secondary atypical meningioma, the present study found that 10-year SMT was 50.1%, comparable to that reported by Koh^67^ (10-year SMT = 49.9%, *n* = 16). By contrast, Krayenbühl et al.^63^ reported mean SMT as 1.95 years in 10 patients with secondary atypical meningiomas, which was much shorter than our result, probably because of the high rate of life-threatening SB tumors (70%) in their study.

### Malignant Transformation in the SR Group

This study observed that benign meningiomas were less likely to be dedifferentiated after SR. Tumors that were treated with SR after surgery, which are generally considered relatively aggressive,^29,50^ had lower IMT (0.82/1000 patient-years) than surgically treated lesions (2.98/1000 patient-years). However, SR-treated meningiomas were more frequently located in the SB than observed in the surgery group. Moreover, SR-treated meningiomas were less frequently treated via salvage surgery, resulting in an unknown histology at progression. Although it is difficult to compare the risk of malignant transformation between the surgery and SR groups, we found no evidence that SR increased the risk.

Because TimeMT tended to be longer in patients treated with radiotherapy than in those treated with surgery alone in individual case analysis, longer follow-up is necessary to clarify the effects of SR. Additionally, imaging studies that can evaluate malignant transformation without surgical intervention would be necessary for a more precise estimation of the incidence of malignant transformation.

## Limitations

This study had several limitations. All adopted studies were retrospective and observational in nature. Although we identified several studies that reported surgical or radiosurgical outcomes for benign meningiomas, few of them reported the number of malignant transformations among their cases. The studies rarely described individual data for malignant changes. Consequently, the evidence level of each study was low. However, the heterogeneity of IMT in the surgery group was largely explained by the tumor location. Conversely, the heterogeneity of IMT in the SR group was not related to the tumor location or gender. We speculated that the SR group might have been more heterogeneous in the initial selection and treatment strategy than the surgery group.

Although Simpson’s grade is considered one of the most important factors for prognosis, we were unable to demonstrate an effect of the extent of surgery on the risk of malignant transformation in this meta-analysis. This was attributable to the lack of information in most studies. To clarify the role of aggressive surgery in the prevention of malignant transformation, further accumulation of data is necessary.

We attempted to pool individual data as much as possible by searching the literature including case reports. The pooled cases might have involved more aggressive tumors because case reports tended to report more aggressive cases with metastases or rhabdoid transformation. However, we found no differences in gender (*P* = .21), age (*P* = .46), tumor location (*P* = .85), time to malignant change (*P* = .34), and survival (*P* = .09) between data from case reports and research articles.

## Conclusion

We found that IMT in benign meningiomas was 2.98/1000 person-years. However, we caution that the increase of malignant transformation may not be constantly linear, especially after 10 years. Furthermore, the rate was considerably affected by the location of meningiomas, as SB tumors had significantly lower IMT. Although IMT was lower in the SR group than in the surgery group, the patient characteristics of the groups differed. Moreover, IMT in the SR group displayed high heterogeneity that was not explained by the tumor location or other clinical factors.

Individual case data revealed that median TimeMT was 5 years, whereas malignant change occurred in patients up to 30 years after the initial surgery. In patients with secondary atypical meningioma, 10-year OS was 50.1%, whereas patients with secondary anaplastic meningioma had a median survival of only 3.1 years.

## Supplementary Material

vdaa129_suppl_Supplementary_Tables

vdaa129_suppl_Supplementary_Figure_1

vdaa129_suppl_Supplementary_File_1

## Notes

**Authorship statement**. Conception and design: S.N., Y.N., and A.N.; data acquisition: S.N., Y.N., and K.N.; data analysis: S.N. and A.N.; data interpretation: S.N., Y.N., A.N., and K.N.; manuscript writing: S.N. and Y.N.

## References

[1] Nakasu S, Nakasu Y Natural history of meningiomas: review with meta-analyses. Neurol Med Chir (Tokyo). 2020;60(3):109–120. 32009127 10.2176/nmc.ra.2019-0213 PMC7073704

[2] Nakasu S, Nakasu Y, Fukami T, Jito J, Nozaki K Growth curve analysis of asymptomatic and symptomatic meningiomas. J Neurooncol. 2011;102(2):303–310. 20686821 10.1007/s11060-010-0319-1

[3] Simpson D The recurrence of intracranial meningiomas after surgical treatment. J Neurol Neurosurg Psychiatry. 1957;20(1):22–39. 13406590 10.1136/jnnp.20.1.22 PMC497230

[4] Champeaux C, Houston D, Dunn L, Resche-Rigon M Intracranial WHO grade I meningioma: a competing risk analysis of progression and disease-specific survival. Acta Neurochir (Wien). 2019. doi:10.1007/s00701-019-04096-9. Online ahead of print. 31707459

[5] Mubeen B, Makhdoomi R, Nayil K, et al. Clinicopathological characteristics of meningiomas: experience from a tertiary care hospital in the Kashmir valley. Asian J Neurosurg. 2019;14(1):41–46. 30937006 10.4103/ajns.AJNS_228_16 PMC6417349

[6] Nakasu S, Fukami T, Jito J, Nozaki K Recurrence and regrowth of benign meningiomas. Brain Tumor Pathol. 2009;26(2):69–72. 19856217 10.1007/s10014-009-0251-2

[7] Hashimoto N, Rabo CS, Okita Y, et al. Slower growth of skull base meningiomas compared with non-skull base meningiomas based on volumetric and biological studies. J Neurosurg. 2012;116(3): 574–580. 22175721 10.3171/2011.11.JNS11999

[8] Voß KM, Spille DC, Sauerland C, et al. The Simpson grading in meningioma surgery: does the tumor location influence the prognostic value? J Neurooncol. 2017;133(3):641–651. 28527009 10.1007/s11060-017-2481-1

[9] Meling TR, Da Broi M, Scheie D, Helseth E Meningiomas: skull base versus non-skull base. Neurosurg Rev. 2019;42(1):163–173. 29627874 10.1007/s10143-018-0976-7

[10] Bi WL, Greenwald NF, Abedalthagafi M, et al. Genomic landscape of high-grade meningiomas. NPJ Genom Med. 2017;2:15. 10.1038/s41525-017-0014-7 PMC5506858 28713588

[11] Gao F, Shi L, Russin J, et al. DNA methylation in the malignant transformation of meningiomas. PLoS One. 2013;8(1):e54114. 23349797 10.1371/journal.pone.0054114 PMC3551961

[12] Champeaux C, Wilson E, Shieff C, Khan AA, Thorne L WHO grade II meningioma: a retrospective study for outcome and prognostic factor assessment. J Neurooncol. 2016;129(2):337–345. 27311726 10.1007/s11060-016-2181-2

[13] Champeaux C, Jecko V, Houston D, et al. Malignant meningioma: an international multicentre retrospective study. Neurosurgery. 2019;85(3):E461–E469. 30566646 10.1093/neuros/nyy610

[14] Yang SY, Park CK, Park SH, Kim DG, Chung YS, Jung HW Atypical and anaplastic meningiomas: prognostic implications of clinicopathological features. J Neurol Neurosurg Psychiatry. 2008;79(5):574–580. 17766430 10.1136/jnnp.2007.121582

[15] Ketter R, Henn W, Niedermayer I, et al. Predictive value of progression-associated chromosomal aberrations for the prognosis of meningiomas_ a retrospective study of 198 cases. J Neurosurg. 2001;95(4):601–607. 11596954 10.3171/jns.2001.95.4.0601

[16] Al-Mefty O, Kadri PA, Pravdenkova S, Sawyer JR, Stangeby C, Husain M Malignant progression in meningioma: documentation of a series and analysis of cytogenetic findings. J Neurosurg. 2004;101(2):210–218. 15309910 10.3171/jns.2004.101.2.0210

[17] Perez-Magan E, Rodriguez de Lope A, Ribalta T, et al. Differential expression profiling analyses identifies downregulation of 1p, 6q, and 14q genes and overexpression of 6p histone cluster 1 genes as markers of recurrence in meningiomas. Neuro Oncol. 2010;12(12):1278–1290. 20685720 10.1093/neuonc/noq081 PMC3018937

[18] Harmancı AS, Youngblood MW, Clark VE, et al. Integrated genomic analyses of de novo pathways underlying atypical meningiomas. Nat Commun. 2018;20(9):16215. 10.1038/ncomms16215 PMC5919704 29676392

[19] Goutagny S, Nault JC, Mallet M, Henin D, Rossi JZ, Kalamarides M High incidence of activating TERT promoter mutations in meningiomas undergoing malignant progression. Brain Pathol. 2014;24(2):184–189. 24261697 10.1111/bpa.12110 PMC8029399

[20] Juratli TA, Thiede C, Koerner MVA, et al. Intratumoral heterogeneity and TERT promoter mutations in progressive/higher-grade meningiomas. Oncotarget. 2017;8(65):109228–109237. 29312603 10.18632/oncotarget.22650 PMC5752516

[21] Jellinger K, Slowik F Histological subtypes and prognostic problems in meningiomas. J Neurol. 1975;208(4):279–298. 50413 10.1007/BF00312803

[22] Jääskeläinen J, Haltia M, Servo A Atypical and anaplastic meningiomas: radiology, surgery, radiotherapy, and outcome. Surg Neurol. 1986;25(3):233–242. 3945904 10.1016/0090-3019(86)90233-8

[23] Schiffer D, Ghimenti C, Fiano V Absence of histological signs of tumor progression in recurrences of completely resected meningiomas. J Neurooncol. 2005;73(2):125–130. 15981101 10.1007/s11060-004-4207-4

[24] McGovern SL, Aldape KD, Munsell MF, Mahajan A, DeMonte F, Woo SY A comparison of World Health Organization tumor grades at recurrence in patients with non-skull base and skull base meningiomas. J Neurosurg. 2010;112(5):925–933. 19799498 10.3171/2009.9.JNS09617

[25] Yeon EK, Sung JY, Do SI, Park BJ, Kim EJ, Na K Clinicoradiological features of recurrent meningioma with high grade transformation. Anticancer Res. 2019;39(11):6299–6305. 31704860 10.21873/anticanres.13840

[26] Evans DG, Birch JM, Ramsden RT, Sharif S, Baser ME Malignant transformation and new primary tumours after therapeutic radiation for benign disease: substantial risks in certain tumour prone syndromes. J Med Genet. 2006;43(4):289–294. 16155191 10.1136/jmg.2005.036319 PMC2563223

[27] Wolf A, Naylor K, Tam M, et al. Risk of radiation-associated intracranial malignancy after stereotactic radiosurgery: a retrospective, multicentre, cohort study. Lancet Oncol. 2019;20(1):159–164. 30473468 10.1016/S1470-2045(18)30659-4

[28] Pollock BE, Stafford SL, Link MJ, Brown PD, Garces YI, Foote RL Single-fraction radiosurgery of benign intracranial meningiomas. Neurosurgery. 2012;71(3):604–612; discussion 613. 22710378 10.1227/NEU.0b013e31825ea557

[29] Santacroce A, Walier M, Regis J, et al. Long-term tumor control of benign intracranial meningiomas after radiosurgery in a series of 4565 patients. Neurosurgery. 2012;70(1):32–39; discussion 39. 21765282 10.1227/NEU.0b013e31822d408a

[30] Celtikci E, Kaymaz AM, Akgul G, Karaaslan B, Emmez OH, Borcek A Retrospective analysis of 449 intracranial meningioma patients operated between 2007 and 2013 at a single institute. Turk Neurosurg. 2018;28(1):1–6. 27593846 10.5137/1019-5149.JTN.17866-16.1

[31] Zulch KJ. Histological Typing of Tumours of the Central Nervous System. Geneva: World Health Organization; 1979.

[32] Kleihues P, Burger PC, Scheithauer BW. Histological Typing of Tumours of the Central Nervous System, 2nd ed. Berlin, Germany: Springer-Verlag; 1993.

[33] Louis DN, Scheithauer BW, Budka H, von Deimling A, Kepes JJ Meningiomas. In: Kleihues P and Cavenee WK, eds. WHO Classification of Tumours of the Central Nervous System. Lyon, France: IARC; 2000.

[34] Perry A, Louis DA, Scheithauer BW, Budka H, von Demling A Meningioma. In: Louis DN, Ohgaki H, Wiestler OD, Cavenee WK, eds. WHO Classification of Tumours of the Central Nervous System. Lyon, France: IARC; 2007:163–172.

[35] Perry A, Louis DN, Budka H, et al. Meningioma. In: Louis DN, Ohgaki H, Wiestler OD, Cavenee WK, eds. WHO Classification of Tumours of the Central Nervous System, revised 4th ed. Lyon, France: IARC; 2016:232–245.

[36] Kanda Y Investigation of the freely available easy-to-use software ‘EZR’ for medical statistics. Bone Marrow Transplant. 2013;48(3):452–458. 23208313 10.1038/bmt.2012.244 PMC3590441

[37] Kojima T, Waga S, Itoh H, Matsubara T, Kuga Y [Clinical analysis of malignant meningiomas]. No Shinkei Geka. 1990;18(10):939–946. 2234295

[38] Chan RC, Thompson GB Morbidity, mortality, and quality of life following surgery for intracranial meningiomas. J Neurosurg. 1984;60:52–60. 6689728 10.3171/jns.1984.60.1.0052

[39] Kalala J-P, Benoit D, de Ridder L Can recurrence of meningiomas be predicted? Anticancer Res. 2004;24:2319–2324. 15330178

[40] Roser F, Samii M, Ostertag H, Bellinzona M The Ki-67 proliferation antigen in meningiomas. Experience in 600 cases. Acta Neurochir (Wien). 2004;146(1):37–44. 14740263 10.1007/s00701-003-0173-4

[41] Fujimoto T, Ishida Y, Uchiyama Y, et al. Radiological predictive factors for regrowth of residual benign meningiomas. Neurol Med Chir (Tokyo). 2011;51(6):415–422. 21701104 10.2176/nmc.51.415

[42] Perez-Magan E, Campos-Martin Y, Mur P, et al. Genetic alterations associated with progression and recurrence in meningiomas. J Neuropathol Exp Neurol. 2012;71(10):882–893. 22964784 10.1097/NEN.0b013e31826bf704

[43] Sahm F SD, Olar A, Koelsche C, et al. TERT promoter mutations and risk of recurrence in meningioma. J Natl Cancer Inst. 2016;108(5):djv377. 10.1093/jnci/djv377 PMC4849806 26668184

[44] Goes P, Santos BFO, Suzuki FS, et al. Necrosis is a consistent factor to recurrence of meningiomas: should it be a stand-alone grading criterion for grade II meningioma? J Neurooncol. 2018;137(2):331–336. 29270884 10.1007/s11060-017-2721-4

[45] Miyagami M, Kanou T, Nakamura S [P53 protein expression and proliferative potential in non-recurrent and recurrent meningiomas]. No to shinkei. 1996;48(8):719–725. 8797205

[46] Stafford SL, Pollock BE Meningioma radiosurgery: tumor control, outcomes, and complications among 190 consecutive patients. Neurosurgery. 2001;49:1029–1038. 11846894 10.1097/00006123-200111000-00001

[47] Torres RC, Frighetto L, De Salles AA, et al. Radiosurgery and stereotactic radiotherapy for intracranial meningiomas. Neurosurg Focus. 2003;14(5):e5. 10.3171/foc.2003.14.5.6 15669816

[48] Malik I, Rowe JG, Walton L, Radatz MW, Kemeny AA The use of stereotactic radiosurgery in the management of meningiomas. Br J Neurosurg. 2005;19(1):13–20. 16147577 10.1080/02688690500080885

[49] Kollová A, Liscák R, Novotný J Jr, Vladyka V, Simonová G, Janousková L Gamma Knife surgery for benign meningioma. J Neurosurg. 2007;107(2):325–336. 17695387 10.3171/JNS-07/08/0325

[50] Seo Y, Kim DG, Kim JW, Han JH, Chung HT, Paek SH Long-term outcomes after gamma knife radiosurgery for benign meningioma: a single institution’s experience with 424 patients. Neurosurgery. 2018;83(5):1040–1049. 29538718 10.1093/neuros/nyx585

[51] Massager N, De Smedt F, Devriendt D Long-term tumor control of benign intracranial tumors after Gamma Knife radiosurgery in 280 patients followed more than 5 years. Acta Neurol Belg. 2013;113(4):463–467. 23709265 10.1007/s13760-013-0211-9

[52] Jang CK, Jung HH, Chang JH, Chang JW, Park YG, Chang WS Long-term results of gamma knife radiosurgery for intracranial meningioma. Brain Tumor Res Treat. 2015;3(2):103–107. 26605265 10.14791/btrt.2015.3.2.103 PMC4656885

[53] Kondziolka D, Patel AD, Kano H, Flickinger JC, Lunsford LD Long-term outcomes after gamma knife radiosurgery for meningiomas. Am J Clin Oncol. 2016;39(5):453–457. 24755664 10.1097/COC.0000000000000080

[54] Hasegawa H, Hanakita S, Shin M, et al. Single-fractionated stereotactic radiosurgery for intracranial meningioma in elderly patients: 25-year experience at a single institution. Oper Neurosurg (Hagerstown). 2018;14(4):341–350. 29554374 10.1093/ons/opx109

[55] Ge Y, Liu D, Zhang Z, et al. Gamma knife radiosurgery for intracranial benign meningiomas: follow-up outcome in 130 patients. Neurosurg Focus. 2019;46(6):E7. 10.3171/2019.3.FOCUS1956 31153153

[56] Cornelius JF, Slotty PJ, Steiger HJ, Hänggi D, Polivka M, George B Malignant potential of skull base versus non-skull base meningiomas: clinical series of 1,663 cases. Acta Neurochir (Wien). 2013;155(3):407–413. 23318687 10.1007/s00701-012-1611-y

[57] Youngblood MW, Duran D, Montejo JD, et al. Correlations between genomic subgroup and clinical features in a cohort of more than 3000 meningiomas. J Neurosurg. 2019:1–10. 10.3171/2019.8.JNS191266 31653806

[58] Yoshida T, Vivatbutsiri P, Morriss-Kay G, Saga Y, Iseki S Cell lineage in mammalian craniofacial mesenchyme. Mech Dev. 2008;125(9–10):797–808. 18617001 10.1016/j.mod.2008.06.007

[59] Savardekar AR, Patra DP, Bir S, et al. Differential tumor progression patterns in skull base versus non-skull base meningiomas: a critical analysis from a long-term follow-up study and review of literature. World Neurosurg. 2018;112:e74–e83. 29258946 10.1016/j.wneu.2017.12.035

[60] Gallagher MJ, Jenkinson MD, Brodbelt AR, Mills SJ, Chavredakis E WHO grade 1 meningioma recurrence: are location and Simpson grade still relevant? Clin Neurol Neurosurg. Feb 2016;141:117–121. 26780494 10.1016/j.clineuro.2016.01.006

[61] Nanda A, Vannemreddy P Recurrence and outcome in skull base meningiomas: do they differ from other intracranial meningiomas? Skull Base. 2008;18(4):243–252. 19119339 10.1055/s-2007-1016956 PMC2467481

[62] Mansouri A, Klironomos G, Taslimi S, et al. Surgically resected skull base meningiomas demonstrate a divergent postoperative recurrence pattern compared with non-skull base meningiomas. J Neurosurg. 2016;125(2):431–440. 26722844 10.3171/2015.7.JNS15546

[63] Krayenbühl N, Pravdenkova S, Al-Mefty O De novo versus transformed atypical and anaplastic meningiomas: comparisons of clinical course, cytogenetics, cytokinetics, and outcome. Neurosurgery. 2007;61(3):495–503. 17881961 10.1227/01.NEU.0000290895.92695.22

[64] Peyre M, Gauchotte G, Giry M, et al. De novo and secondary anaplastic meningiomas: a study of clinical and histomolecular prognostic factors. Neuro Oncol. 2018;20(8):1113–1121. 29216385 10.1093/neuonc/nox231 PMC6280137

[65] Zhao P, Hu M, Zhao M, Ren X, Jiang Z Prognostic factors for patients with atypical or malignant meningiomas treated at a single center. Neurosurg Rev. 2015;38(1):101–107. 25139398 10.1007/s10143-014-0558-2

[66] Champeaux C, Wilson E, Brandner S, Shieff C, Thorne L World Health Organization grade III meningiomas. a retrospective study for outcome and prognostic factors assessment. Br J Neurosurg. 2015;29(5):693–698. 26098606 10.3109/02688697.2015.1054350

[67] Koh EJ Malignant progression of meningioma: a retrospective observational study http://hdl.handle.net/10371/137988. 2017. Accessed February 6, 2020.

