# Supplementary material for: Malignant transformation of WHO grade I meningiomas after surgery or radiosurgery: systematic review and meta-analysis of observational studies
Source: Neurooncol Adv. 2020 Oct 16;2(1):vdaa129. doi: 10.1093/noajnl/vdaa129 (PMC7712809; doi:10.1093/noajnl/vdaa129)
Supplement: vdaa129_suppl_Supplementary_Figure_1 [file vdaa129_suppl_supplementary_figure_1.docx]

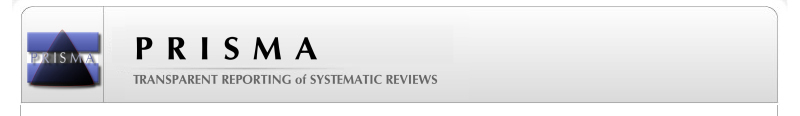
**Supplementary Figure**

**Figure 1. PRISMA 2009 Flow Diagram**

**PubMed Search** 1979-2019 **&** Meningioma* **Ti/Ab &** Human **&** age > 19y.o. **= 10205**

**NOT** neurofibromatosis **[Title] = 10076**

**NOT** ectopic**[Title] OR** diploic**[Title] OR** intraosseous**[Title] OR** "optic nerve sheath**"[Title] OR** primary pulmonary**[Title] = 9676**

**AND (transformation OR dedifferentiation OR "malignant change" OR progression OR recurrence)**

**= 2342**

Full-text articles assessed for eligibility
(n = 904)

## Included

Records screened by title and abstract (n = 3989)

Studies included; meta-analysis)(n = 24 ); individual data analysis (n = 84 + 9 from meta-analysis)

Studies included by hand-search and cross-referencing (n = 12)

## Eligibility

Records excluded

(n = 3085)

Full-text articles excluded,
(n = 808)

Records screened title (n = 196)

Records identified through Google Scholar
(n = 888)

Records identified through

PubMed database (n = 2342), Scoups (n = 3181), Cochrane library (n = 0), Japan Medical Abstract Society (n = 107)

Total （n = 5826）

Records after duplicates removed
(n = 3989)

## Screening

## Identification

Studies included in analysis
(n = 96)
